# Comparative Three‐Barcode Phylogenetics and Soil Microbiomes of Planted and Wild *Arbutus* Strawberry Trees

**DOI:** 10.1002/pld3.70078

**Published:** 2025-05-08

**Authors:** Flannery McLamb, Armando Vazquez, Natalie Olander, Miguel F. Vasquez, Zuying Feng, Niharika Malhotra, Liisa Bozinovic, Karen Najera Ruiz, Katherine O'Connell, Joseph Stagg, Goran Bozinovic

**Affiliations:** ^1^ Boz Life Science Research and Teaching Institute La Jolla California USA; ^2^ Division of Extended Studies University of California San Diego La Jolla California USA; ^3^ Hope College Holland Michigan USA; ^4^ Oregon Bioscience Association Portland Oregon USA; ^5^ Bowdoin College Brunswick Maine USA; ^6^ Portland State University Portland Oregon USA; ^7^ Pharos International Institute for Science, Arts and Culture Stari Grad Croatia; ^8^ School of Biological Sciences University of California San Diego La Jolla California USA

**Keywords:** 16S rRNA, biodiversity, matK, metagenomics, phylogeny, rbcL, soil chemistry, trnH‐psbA

## Abstract

Taxonomic identification of closely related plants can be challenging due to convergent evolution, hybridization, and overlapping geographic distribution. To derive taxonomic relationships among planted and wild *Arbutus* plants across a large geographic range, we complemented three standard plastid barcodes *rbcL*, *matK*, and *trnH‐psbA* with soil and fruit chemistry, soil microbiome, and plant morphology analyses. Soil and plant sampling included planted *Arbutus* from manicured sites in Southern California, USA, wild plants from Southern and Northern California, and wild populations from Mediterranean island of Hvar, Croatia. We hypothesized that phenotypic variation within and between sites correlates with plants' genotype and geographic distribution. Similar fruit chemistry corresponds to geographical proximity and morphological resemblance, while bulk soil bacterial content defines three distinct clusters distinguishing planted versus wild trees and continent of origin. The soil microbiome of wild California *Arbutus* was characterized by an abundance of *Nitrobacter*, while the presence of *Candidatus Xiphinematobacter* was high in wild Hvar samples and most planted samples, but low in all wild California samples. Although all three barcodes resolved four main groups, the position of samples varies across barcodes. The *rbcL* phylogram is relatively unbalanced, suggesting slower diversification among wild California populations and exhibiting greater resolution than other barcodes among planted individuals. While our data demonstrate an overall agreement among standard plant barcodes relative to geo‐distribution and plant morphology, sustained efforts on cost‐effective global plant DNA barcode library standardization for closely related and geographically overlapping plants is recommended.

## 1 Introduction

In *Naturalis Historia*, Pliny the Elder describes how the strawberry tree, *Arbutus unedo* , was named *unedo* as people could “only eat one” (“*unum edo*”) of its berries, likely due to an unpleasant or bland taste (Rackham 1960). *A. unedo* , native to the Mediterranean Basin and Western Europe (Sealy 1949), is distributed globally. They are popular ornamental plants with attractive flowers and fruits and are easily cultivated once established (Celikel et al. 2008; Lopes et al. 2012; Mhamdi Takrouni et al. 2012). Their fruits and leaves have compounds with antioxidant activities (Mendes et al. 2011; Oliveira et al. 2011), and the fruits have been used for spirit production (Galego et al. 2013; Botelho et al. 2015). *Arbutus* are flowering evergreen shrubs or small trees in the family *Ericaceae*. Although their fruits are somewhat similar, *Arbutus* and *Fragaria* (strawberries) are not closely related genera. *Arbutus* genus members are distributed notably along the North America western coast via the Madrean–Tethyan dispersion hypothesis (Axelrod 1975; De Santis et al. 2024). The cool, wet summers of the United Kingdom and temperate climates of Europe and Asia are also conducive to the paraphyletic *Arbutus* genus; among those from the Mediterranean Basin, *A. unedo* , *Arbutus andrachne* , and *Arbutus canariensis* are closely related (Hileman et al. 2001). *Arbutus* × *andrachnoides* (syn. *A*. × *hybrida*, or *A. andrachne* × *unedo*) is a naturally occurring hybridization between *A. unedo* and *A. andrachne* (Bertsouklis and Papafotiou 2016) that is popularly sold as an ornamental plant under the name Arbutus “Marina.” Because it is a hybrid, its seeds are unlikely to produce true‐to‐type offspring (Curry 2023). Accurate identification of these closely related species is challenging due to hybridization, speciation, and overlapping geographic distribution.

DNA barcodes were developed for accurate, efficient identifications of biological samples. Ideally, these standardized sequences of roughly 400–800 base pairs are easily sequenced (Hebert et al. 2003), ultimately yielding a reference database for thousands of diverse plant species (Cowan et al. 2006). DNA barcoding is a relatively quick and affordable tool for identifying species within and between populations with accessible plant tissues such as leaves and stems when other options are impractical. However, it lacks resolution because low taxonomic levels may only differ in sequences outside of selected barcoding regions (Dong et al. 2012). Commonly used plant barcodes include *rbcL*, *matK*, and *trnH‐psbA*, which have furthered biologists' understanding of ecology and evolutionary biology (Tripathi et al. 2013; Bieniek et al. 2015; Cabelin and Alejandro 2016). These barcodes have been proposed as a standard combination: *rbcL* primers are widely applicable across clades, while *matK* and *trnH‐psbA* have higher resolution but lower primer universality and sequence length consistency, respectively (CBOL Plant Working Group 1 et al. 2009). The effectiveness of DNA barcoding also varies between taxa (Spooner 2009). For example, *rbcL* and *matK* failed to resolve species from the *Berberis*, *Ficus*, and *Gossypium* genera (Roy et al. 2010), while Ericaceae, the family of *Arbutus*, exhibited the fourth highest *rbcL* resolution and the sixth highest *matK* resolution among 25 vascular plant families in Canada (Braukmann et al. 2017). Ericaceae genera were among the top 12%, 11%, and 16% accurately identified by *rbcL*, *matK*, and *trnH‐psbA* sequences from NCBI GenBank, respectively (Kolter and Gemeinholzer 2021). Highly variable morphology and poorly defined species boundaries challenge accurate species identification (Fazekas et al. 2008), particularly among woody taxa like *Arbutus* with long generation times, and in closely related populations with few distinct genetic sequences. To our knowledge, barcoding has not been performed on closely related *Arbutus* individuals from two continents. Because resolution and accuracy increase when analyzing more characters or barcodes, we investigated whether the three standard plastid barcodes (*rbcL*, *matK*, and *trnH‐psbA*) in combination would be sufficient to resolve close or distant relationships in *Arbutus* trees. Planted and wild populations distributed across California, USA, and Island of Hvar, Croatia, vary in geographic location, morphology, evolutionary history, and dispersal over time. We expected location, morphological, and genetic similarity to be associated, and therefore evident in the combined three barcodes' phylogeny. Such plastid DNA barcoding can generate informative molecular relatedness data, providing the foundation for more comprehensive and sensitive methods. For instance, microsatellites and RAD‐seq have been used to complement plastid barcoding in other plant taxa (Scariolo et al. 2021; Terrones et al. 2022).

The plant–soil interaction plays an important role in plant evolution (Lau and Lennon 2011; Schweitzer et al. 2014; Hawkes et al. 2020). Chemicals and microbes in the soil can critically impact the nutrition, development, and immune response of associated plants (Carvalhais et al. 2013; Sahu et al. 2019; Korenblum et al. 2020; Panda and Das 2024). The effect of plant evolution and physiology on associated microbial communities is largely unknown. We therefore supplemented our phylogeny results with 16S rRNA sequencing of bulk soil bacteria and soil chemistry. Geographic location, topography, rainfall, and drought can shift microbe diversity and abundance, and soil chemistry both seasonally and permanently (Cleveland et al. 2004; Drenovsky et al. 2010; Sharma and Gobi 2016; Siles et al. 2016). Because environmental stressors influence plant‐specific host–microbe coadaptative mechanisms (Matveeva et al. 2018; Hassani et al. 2019), we expected differing conditions and stressors between planted and wild populations to further differentiate population‐specific microbial composition.

Plant morphology and fruit chemistry were also used to better understand variation within and between populations. *A. unedo* matures in about a year and grows as either shrubs or trees that are generally 4–7 m tall but can reach up to 15 m tall with an 80 cm trunk diameter. Its outer layer of bark is reddish and smooth, but peels to reveal a lighter brown layer. Its green leaves are 8–10 cm long and 3–4 cm broad, with a glossy upper side and dull underside. Pinkish‐white, bell‐shaped flowers grow 7–8 mm in diameter in autumn and smell mildly sweet. Its fruit somewhat resembles a strawberry, but it is distinct. Round yellow‐to‐red berries are 7–20 mm in diameter and covered in soft spikes, with a sweet taste when ripe (Sealy 1949; Flora of North America Editorial Committee 2009; Tartaglia et al. 2023).

**FIGURE 1:**
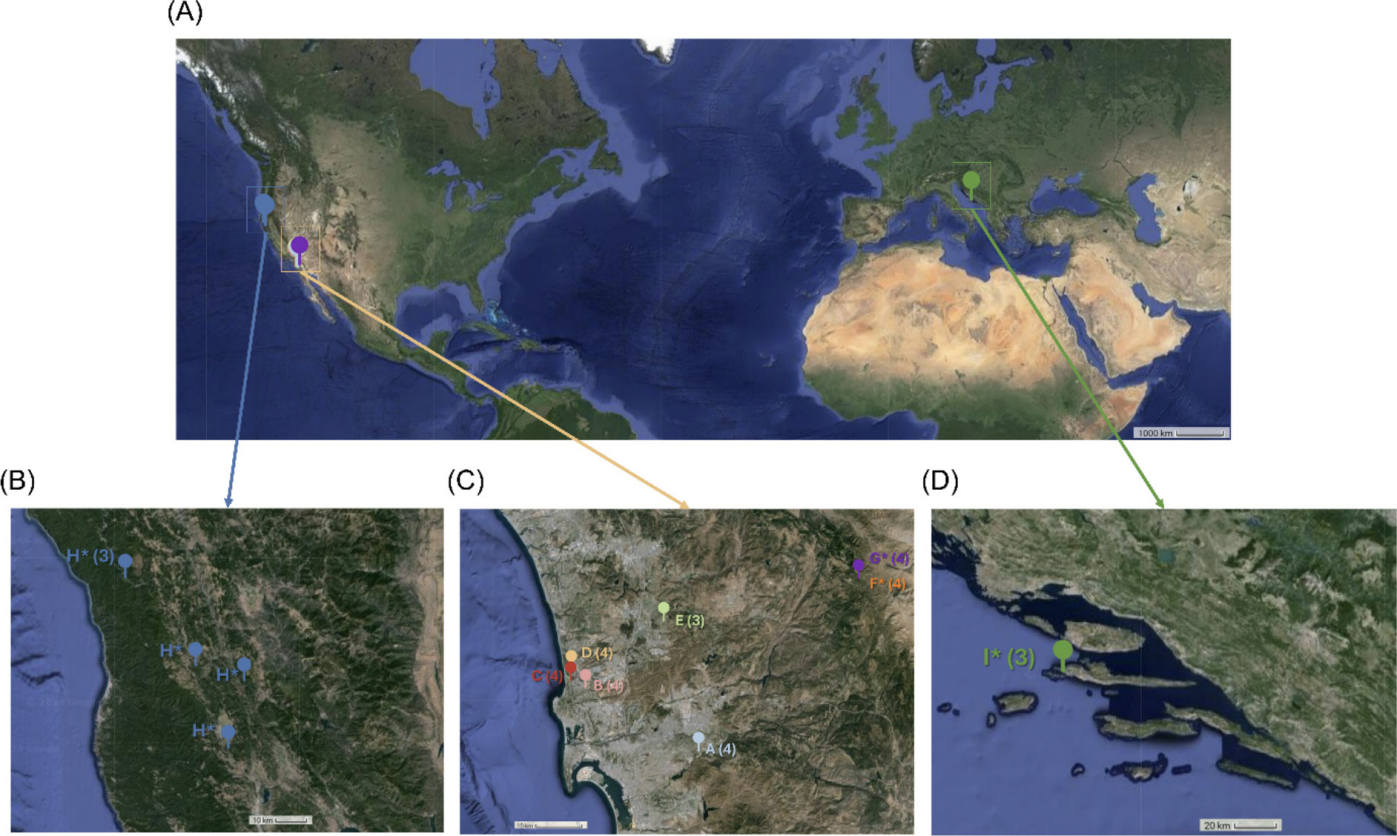
*Arbutus* samples were collected from (A) sites in USA and Croatia: (B) Northern California, USA, (C) Southern California, USA, and (D) Hvar, Croatia. Pins and letters indicate sampling sites (Table 1), and numbers indicate the number of sampling locations. For example, A (4) indicates four sampling locations at the sampling site A. Because the sample locations are significantly more distant at the Northern California site H*, nonparenthetical numbers denote exact sample locations (Table 1). Asterisks denote wild trees, relative to planted urban trees.

We hypothesized that phenotypic variation would correlate with plants' genotype and geographic distribution (Figure 1): planted, concentrated urban trees and their soil would show the least variation in soil chemistry and microbiome composition, display almost uniform tree, leaf, and fruit morphology and fruit chemistry, and result in relatively low within‐group sequencing variation for all three barcodes. As the geographical distance expands to southern and northern mountainous areas of California, the phenotypes and barcode sequence variation would increase; notably, the natural population on the island of Hvar, Croatia would have the lowest variance among wild populations due to isolation and smaller sample size but still display a larger variation than closely clustered, planted urban California trees. We collected individual plants and associated soil accordingly, per an approximately 10‐fold increase in tree distance within the site: planted trees and their soil were sampled from three concentrated urban San Diego, California clusters within a 5.1‐km radius, with the individual plant sample distance of about 50 m within the sampling cluster. To test for reduced genetic and phenotypic variations among the trees on the Croatian island of Hvar relative to those in California, wild plants and associated soil from the island of Hvar were sampled within an approximately 500‐m radius. The distribution of one southern and two Northern California wild population sites were chosen randomly from densely forested areas, including the samples in Northern California that were approximately 65 km apart. Along with plant morphology, soil microbiome and composition, and fruit chemistry, we utilized standard barcodes to genetically relate a recently evolved *Arbutus* species relative to older native Croatian island populations and infer their genetic relatedness. We use three standard barcodes *rbcL*, *matK*, and *trnH‐psbA* to distinguish planted and wild populations concentrated in coastal Southern California, wild populations from inland Northern California, and wild populations from a Mediterranean island in Croatia.

## 2 Materials and Methods

### 2.1 Sample Selection

Thirty‐five trees tentatively identified as *Arbutus unedo* (Figure 1) were sampled across eight sites in California, USA, and one site on the Island of Hvar, Croatia, which include planted (sites A–E) and wild (sites F*–I*) populations. We define planted populations as ornamental trees deliberately planted within urban, densely populated areas, and wild populations as trees spontaneously grown in nonurban settings without upkeep from humans. Site details are provided in Table 1.

**TABLE 1:** Leaf, fruit, and soil *Arbutus* sampling sites.

Sampling site	Sample	Coordinates	Date sampled	Number of soil replicates	Number of trees in pooled fruit sample
College campus in San Diego, California	A1	32.744497, −116.944143	2/2/2021	1	3
	A2	32.744497, −116.944143	7/3/2021	3	
	A3	32.744519, −116.943832	2/2/2021	1	
	A4	32.744519, −116.943832	7/3/2021	3	
Residential area in San Diego, Southern California	B1	32.8664901, −117.2055304	6/18/2021‡	3	2
	B2	32.8664901, −117.2055304	6/18/2021‡	3	
	B3	32.8664966, −117.2056477	6/18/2021‡	3	
	B4	32.8665146, −117.2055944	6/18/2021‡	3	
University campus in San Diego, Southern California	C1	32.8756336, −117.2403552	1/23/2021	3	3
	C2	32.8820140, −117.2407377	1/23/2021	3	
	C3	32.8823017, −117.2407417	1/23/2021	3	
	C4	32.8823017, −117.2407417	1/23/2021	3	
Office building parking lot in San Diego, Southern California	D1	32.9039323, −117.238976	1/23/2021	2	2
	D2	32.9039323, −117.238976	1/23/2021	2	
	D3	32.9039584, −117.2390451	1/23/2021	2	
	D4	32.9039584, −117.2390451	1/23/2021	2	
High school campus near San Diego, Southern California	E1	32.998088, −117.024749	10/5/2022†	1	Not analyzed
	E2	32.998126, −117.024704	10/5/2022†	1	
	E3	32.998164, −117.024745	10/5/2022†	1	
Roadside area in Julian, Southern California (northeast of San Diego)	F1*	33.065291, −116.5834631	5/8/2022	2	3
	F2*	33.065291, −116.5834631	5/8/2022	2	
	F3*	33.065291, −116.5834631	5/8/2022	3	
	F4*	33.065291, −116.5834631	5/8/2022	1	
Mountainous area in Julian, Southern California (northeast of San Diego)	G1*	33.0808056, −116.5730556	6/18/2021	3	3
	G2*	33.0808056, −116.5730556	6/18/2021	3	
	G3*	33.080941, −116.573248	3/3/2021	1	
	G4*	33.080941, −116.573248	3/3/2021	1	
Forested areas in Northern California	H1*	39.3539483, −123.32015	6/29/2022	1	3
	H2*	39.5506383, −123.258835	6/28/2022	1	
	H3*	39.5961306, −123.44116	6/28/2022	1	
	H4*	39.853115, −123.7099867	6/28/2022	1	
	H5*	39.8535367, −123.7096767	6/28/2022	1	
	H6*	39.8535383, −123.7096767	6/28/2022	1	
Island of Hvar	I1*	43.177339, 16.425147	8/20/2021	1	Not pooled; each tree's fruit was analyzed separately to assess diversity
	I2*	43.1798694, 16.4240111	8/20/2021	1	
	I3*	43.1814361, 16.4206583	8/20/2021	1	

*Note:* The sites are ordered by geographical distribution from south (site A) to north (site I*); sampling locations within the sites were also ordered from south to north (1–6), so that the most southern sampling location is A1, and the most northern sampling location is I3*. The asterisks (*) denote the *Arbutus* wild trees, relative to planted urban trees. †Soil collected separately on 3/17/2023. ‡Fruit collected separately on 5/14/2021.

### 2.2 Soil and Fruit Composition

Three soil samples within 10 cm of the base of each tree (Table 1; *n* = 36) were collected at a maximal depth of 30 cm using aluminum coring devices cleaned with 70% EtOH between each collection; soil around Hvar plants were collected in the same manner using stainless steel large common spoon utensils. Soil samples were pooled and manually homogenized per each tree, transported in 1‐L sampling plastic bags and stored at −20°C. Soil samples of 100–150 g were analyzed for cation exchange capacity (CEC), percent base saturation, metallic elements, and phosphorus using Mehlich I extraction (Kissel and Sonon 2008; Sonon et al. 2022), lime buffer capacity (LBC) and equilibrium lime buffer capacity (LBCeq) by Ca(OH)_2_ titration (Sonon et al. 2015), pH values in a 1:1 soil to 0.01‐M CaCl_2_ solution, and total organic carbon (TOC) using a Shimadzu TOC‐5050A Total Organic Carbon Analyzer (Kissel and Sonon 2008). For each US site except E, approximately 50 g of fruit collected and stored at −20°C were pooled and analyzed for moisture, crude fiber and protein through wet chemistry, metallic elements, and phosphorous (Plank and Kissel 2008). Hvar fruits were processed in the same manner, except that fruit from each tree was analyzed separately. Soil and fruit chemistry were analyzed by the Agricultural and Environmental Services Laboratories, University of Georgia, USA. Fruit chemistry data were visualized in GraphPad Prism (version 9.3.1 for Windows, GraphPad Software, San Diego, California, USA, www.graphpad.com) with Ward's hierarchical clustering in R (version 4.3.1) (R Core Team 2024), and soil chemistry hierarchical clustering and heatmap were generated in JMP Pro (version 14.3.0., SAS Institute Inc., Cary, NC, 1989–2023).

### 2.3 Leaf Tissue DNA Barcoding

Multiple leaves per tree were collected by stem or from separate branches and stored at −20°C until DNA extraction. Approximately 100 mg of wet leaf tissue were flash frozen with liquid nitrogen and homogenized using a manual mortar and pestle, or Bel‐Art SP Scienceware Disposable Polypropylene Pestles (catalog number: F199230001, Bel‐Art, Wayne, New Jersey, USA) attached to a DEWALT Bare‐Tool DCD760B 1/2‐Inch 18‐Volt Cordless Compact Drill/Driver (catalog number: DCD760, Dewalt, Towson, Maryland, USA) in 400 μL of Buffer AP1 and 4 μL of RNase A stock solution from the DNeasy Plant Mini Kit (catalog number: 69104, QIAGEN, Hilden, Germany) for low DNA yield samples.

Total leaf DNA was extracted using the DNeasy Plant Mini Kit (catalog number: 69104, QIAGEN, Hilden, Germany). Barcoding regions of *rbcL*, *matK*, and *trnH‐psbA* were amplified in an Axygen Maxygene II Thermal Cycler (catalog number: THERM‐1001, Axygen a Corning brand, New York, USA) using GoTaq Green Master Mix (catalog number: M7122, Promega, Madison, Wisconsin, USA). Primers were generated using Primer3: *rbcL*‐F 5′‐TCG TTA CAA AGG GCG ATG CT‐3′, *rbcL*‐R 5′‐TGT CCT AAA GTT CCG CCA CC‐3′, *matK*‐F 5′‐GGA TTT GCA GTC GTT GTG GA‐3′, *matK*‐R 5′‐ACG CCC GAA TCG GTC AAT AA‐3′, *trnH‐psbA*‐F 5′‐GTT ATG CAT GAA CGT AAT GCT C‐3′, *trnH‐psbA*‐R 5′‐CGC CGA TGG TGG ATT CAC AAA TC‐3′ (Tate and Simpson 2003). Sanger sequencing was performed by Eton Bioscience Inc. and Genewiz from Azenta Life Sciences using ABI‐3730 xl DNA analyzer.

DNA sequences and chromatograms were visualized in SnapGene software (version 6.1.1; www.snapgene.com) for quality control. Sequences were trimmed via sangeranalyseR (version 1.8.0) (Chao et al. 2021) using the M2 method with a cutoff quality score of 30 and a sliding window size of 10. Sequences for each *Arbutus* DNA barcode were entered individually into the NIH's standard nucleotide Basic Local Alignment Search Tool (BLAST) (Altschul et al. 1990) using the standard nucleotide collection database and optimized for highly similar sequences (megablast).

Respective barcoding sequences of *Glycine max* , *Zea mays* , *Oryza sativa* , and *Arabidopsis thaliana* were gathered from NCBI (Table S1) and the *Arbutus* trimmed sequences for each barcode were aligned via the ClustalW method using msa (version 1.30.1) (Bodenhofer et al. 2015). Maximum likelihood phylogenetic trees with bootstrap values were assembled in MEGA (version 10.2.6) (Kumar et al. 2018) according to the model test for *rbcL*, *matK*, and *trnH‐psbA* individually and as a concatenation. Tanglegrams were visualized using the dendextend package (version 1.17.1) (Galili 2015) in R, and the untangle function was used to optimize tanglegram layouts. Individual tanglegrams were combined using Figma (version 116.12.2; www.figma.com). Tree similarity and distance were quantified using mutual clustering metrics (Smith 2020) and branch scores (Kuhner and Felsenstein 1994), implemented in R via TreeDist (version 2.7.0) (Smith 2019) and ape (version 5.7‐1) (Paradis and Schliep 2019), respectively.

### 2.4 16S rRNA Soil Microbial Sequencing and Analysis

DNA was extracted from soil samples using the DNeasy PowerSoil Pro Kit (catalog number: 47014, QIAGEN, Hilden, Germany). Library preparation was performed using the NEXTflex™ 16S V4 Amplicon‐Seq Kit 2.0 (catalog number: NOVA‐4203‐03, Revvity, Waltham, MA, USA). Libraries were quantified with a 2100 Bioanalyzer Instrument (Agilent Technologies, Santa Clara, CA, USA), 2400 TapeStation System (catalog number: G2991BA, Agilent Technologies, Santa Clara, CA, USA) and a Qubit 4 Fluorometer (ThermoFisher Scientific, Waltham, MA, USA). Samples were pooled in a NextSeq™1000/2000 P1 Reagents (600 cycles) flow cell (catalog number: 20075294, Illumina, San Diego, California, USA) for 2.5 million reads per sample for 36 samples. Demultiplexed 16S rRNA sequences were imported and analyzed using DADA2 (version 1.28.0) (Callahan et al. 2016) in R. Trimming and filtering parameters were set as default except for truncLen = c(300,210), and maxEE = c(2,2): to keep mean base position Phred scores above 30, forward and reverse reads were trimmed to 300 and 210 base pairs, respectively, and the maximum expected errors were two per read. Default parameters were used for modeling error rates, inferring sequence variants, dereplication, and merging paired‐end reads. Chimeras were removed via the consensus method, and the remaining sequences were assigned taxonomies via the SILVA 138.2 reference database (Quast et al. 2013; Yilmaz et al. 2014). Sample read counts after sequencing, filtering, merging paired‐end reads, and removing chimeras are in Table S2. Shannon and Simpson alpha diversity, Curtis–Bray nonmetric multidimensional scaling (NMDS), and bar plots were analyzed and generated using phyloseq (version 1.44) (McMurdie and Holmes 2013). Analyses of variance (ANOVAs) on distance matrices were conducted using adonis2 in the vegan package (version 2.6‐4) (Oksanen et al. 2022).

## 3 Results

### 3.1 Arbutus Tree and Fruit Morphology

**FIGURE 2:**
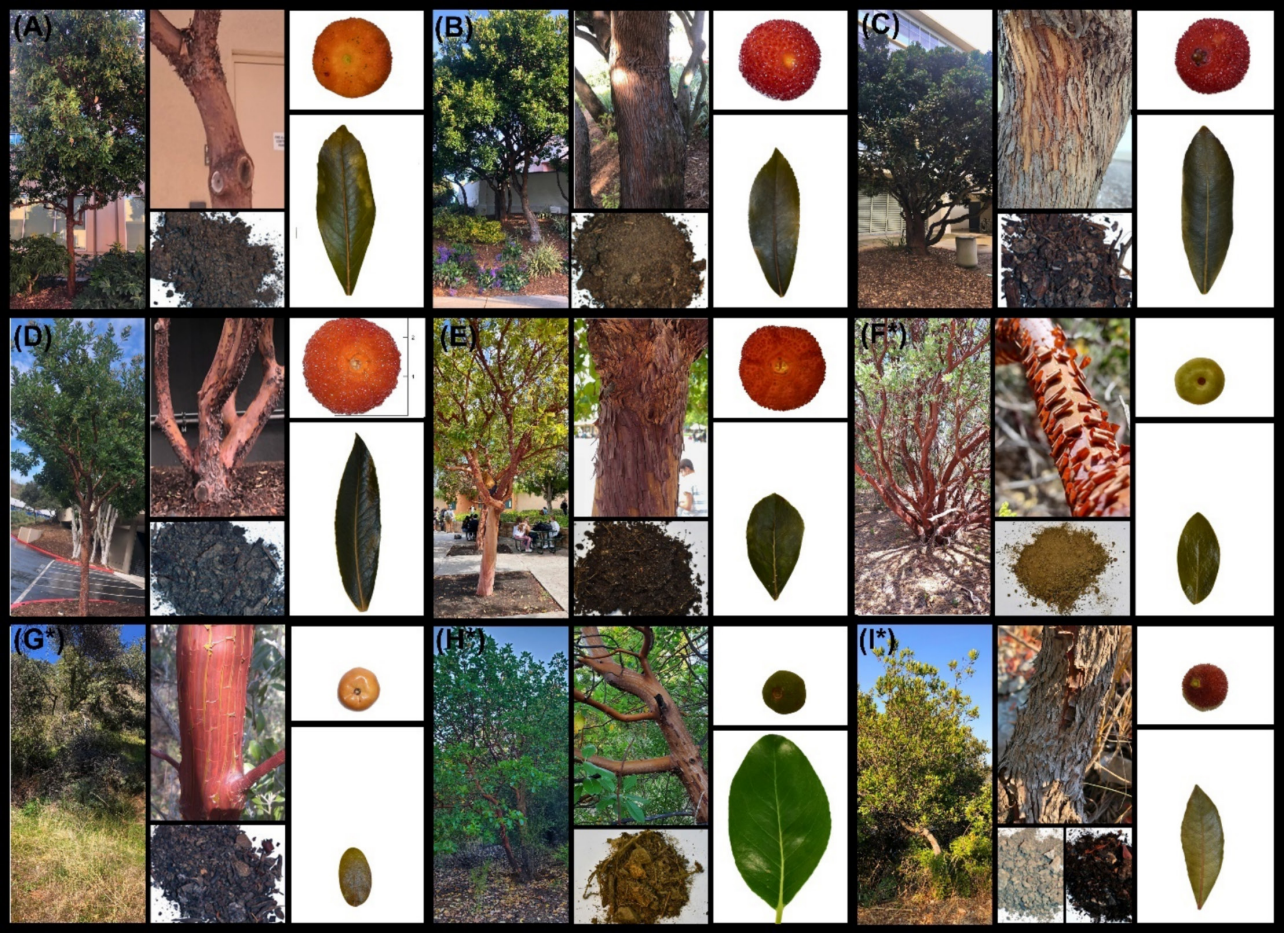
Images within each panel from left to right and top to bottom show tree, bark, soil, fruit, and leaf from representative individuals at each site.

Most of the *Arbutus* morphological phenotypes share consistent characteristics of reddish smooth bark and elliptic or oblong dark‐green leaves with serrulated margins (Flora of North America Editorial Committee 2009). There are significant differences between planted and wild populations (Figure 2): planted, maintained trees from Southern California sites (A–E) have a main tree trunk, well defined relatively dense tree canopy, larger white bell‐shaped flower clusters, and bumpy yellowish‐red mature fruit. Wild *Arbutus* trees in Southern California (F* and G*) are shorter and characterized by more branches stemming from the main trunk closer to the tree root, narrower and less leaf‐dense canopies, and fruit clusters with smaller, yellowish or light green, smooth‐skinned fruit (Figure 2). Hvar *Arbutus* trees (I*) look very similar to Southern California wild population trees, but have small, bumpy dark red fruit. Northern California trees are the tallest among those sampled, with broad canopies and small, smooth, dark green fruit.

### 3.2 Fruit Chemistry

**FIGURE 3:**
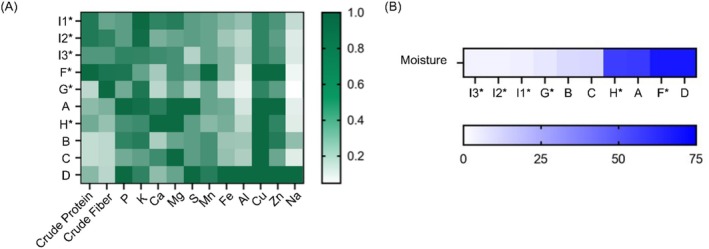
Chemical characteristics of fruit by sampling site. (A) Rows represent hierarchically clustered sampling sites and columns indicate chemical characteristics of fruit as dry matter. Data were normalized to the highest value for each characteristic, with darker green indicating higher relative values. Raw data are in Table S3. (B) Fruit moisture content from pooled fruit samples was determined as a percentage of raw fruit. Darker blue indicates higher moisture content and samples are ordered from lowest to highest moisture.

Crude protein was highest in site F* fruit (4.8%) and lowest in sites B and C fruit (1.3%) (Figure 3A). Site G* had the highest crude fiber at 46.7% while site B had the lowest at 13.9%. Macronutrients (phosphorus, potassium, calcium, magnesium, and sulfur) were highest in site A fruits (0.12%, 0.90%, 0.31%, 0.08%, and 0.03%), except for higher potassium in I2* and I1* samples (0.91% and 0.93%, respectively) and higher calcium in site H* (0.36%). Macronutrients were lowest in site G* (0.07%, 0.85%, 0.11%, 0.05%, and 0.01%) except for lower potassium in site F*. Of the other tested nutrients (manganese, iron, aluminum, copper, zinc, and sodium), site D had most of the highest levels (7, 76, 90, 6, 18, and 1110 ppm) except for manganese in site F*. Site G* had most of the lowest levels (< 5, 19, 11, < 5, 12, and < 54 ppm; Figure 3A), except for zinc in site C. Fruit moisture, ranging from 3.4% to 68.4%, was the lowest in site I* samples and highest in site D samples. Site I*samples, which were not pooled, had relatively similar moisture levels across all three samples (3.4%–4.5%; Figure 3B).

### 3.3 Soil Chemical Analysis Distinguishes Between Manicured and Wild Sampling Sites

**FIGURE 4:**
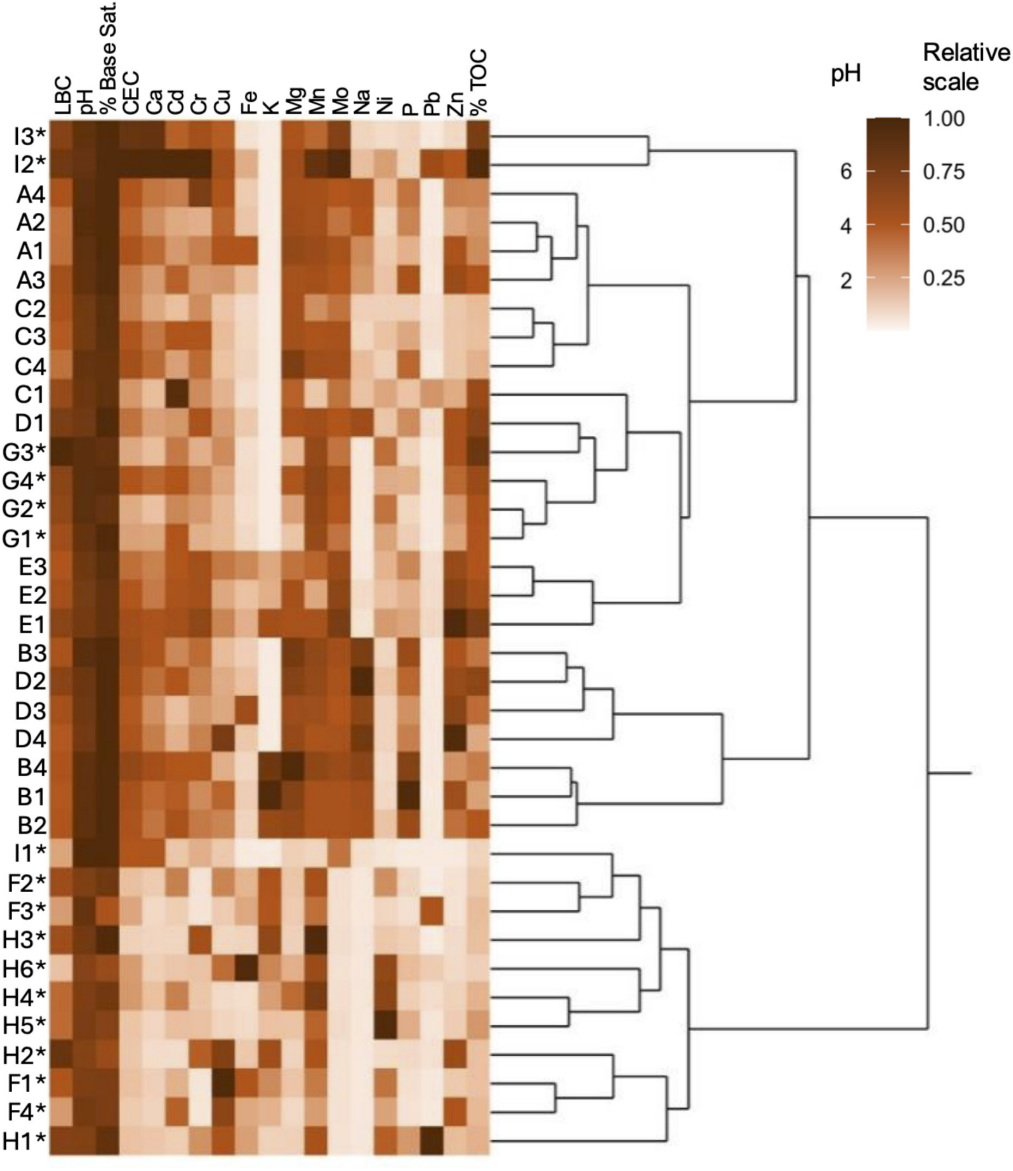
Hierarchical clustering of samples by soil chemistry characteristics. Rows represent pooled soil samples of each tree, and columns correspond to chemical characteristics. Darker colors represent higher relative values. Dendrogram branch lengths are proportional to similarities in chemical characteristics. Raw data are in Table S4.

Hierarchical clustering of soil samples by chemical characteristics revealed four main clusters: site I*; sites A, C, E, and G*; sites B and D; sites F* and H* (Figure 4). Within clusters, samples are largely grouped by site. Samples outside their respective clusters included D1 grouping within cluster A/C/E/G* and I1* grouping in F*/H*. Geographically adjacent sampling sites (B‐D) and several close‐lying samples (Figure 1) within sites (D1–2; I1–3*; B3–4) grouped separately (Figure 4).

### 3.4 Soil Microbial Composition Varies Between Wild and Urban Sites by Continent

**FIGURE 5:**
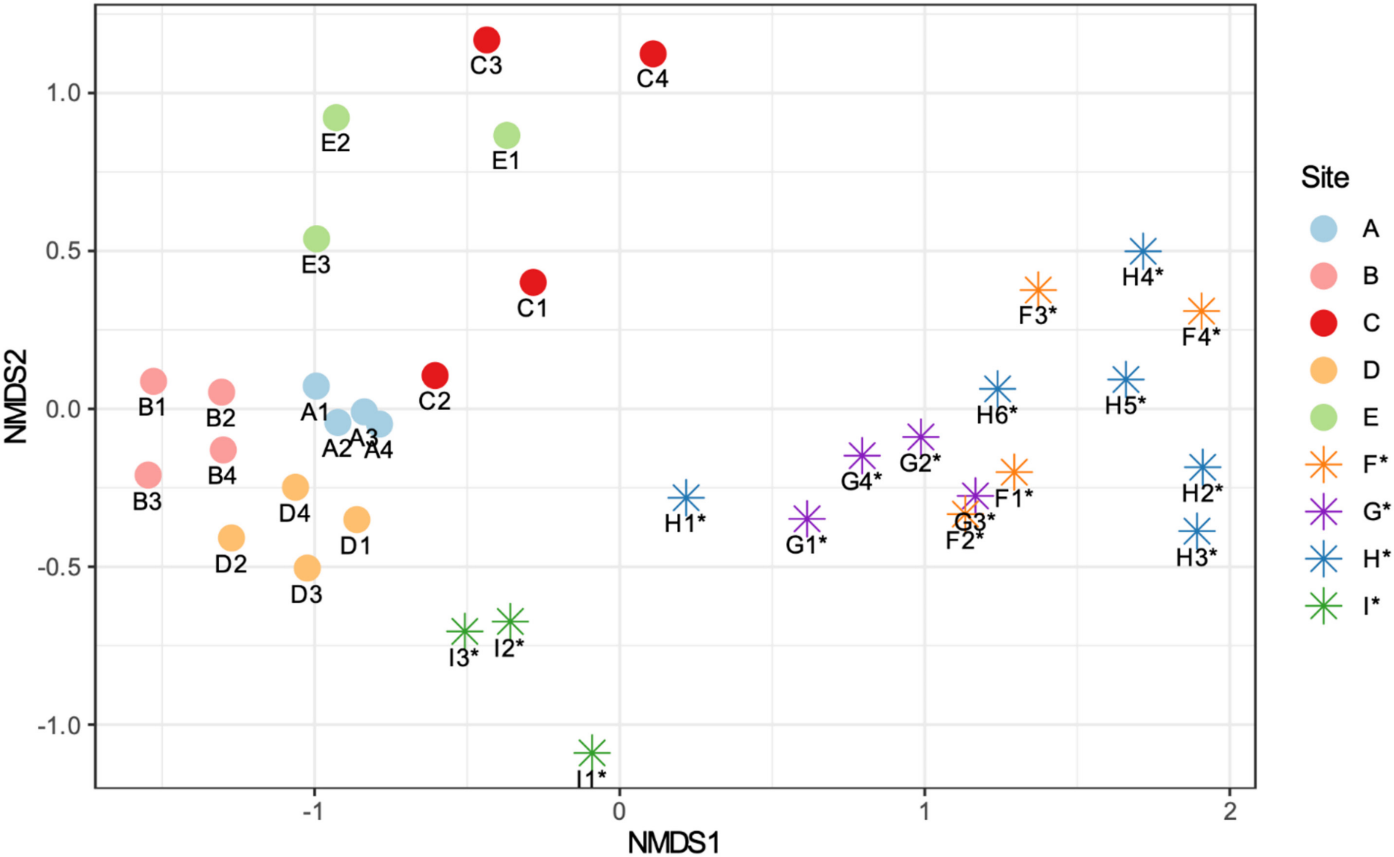
Nonmetric multidimensional scaling (NMDS) based on Bray–Curtis similarity showing 16S rRNA taxonomic profiles by soil sample. Each point represents the soil microbial composition at an individual tree, where points that are close together have similar compositions. Colors indicate site. Stress is 0.083.

**FIGURE 6:**
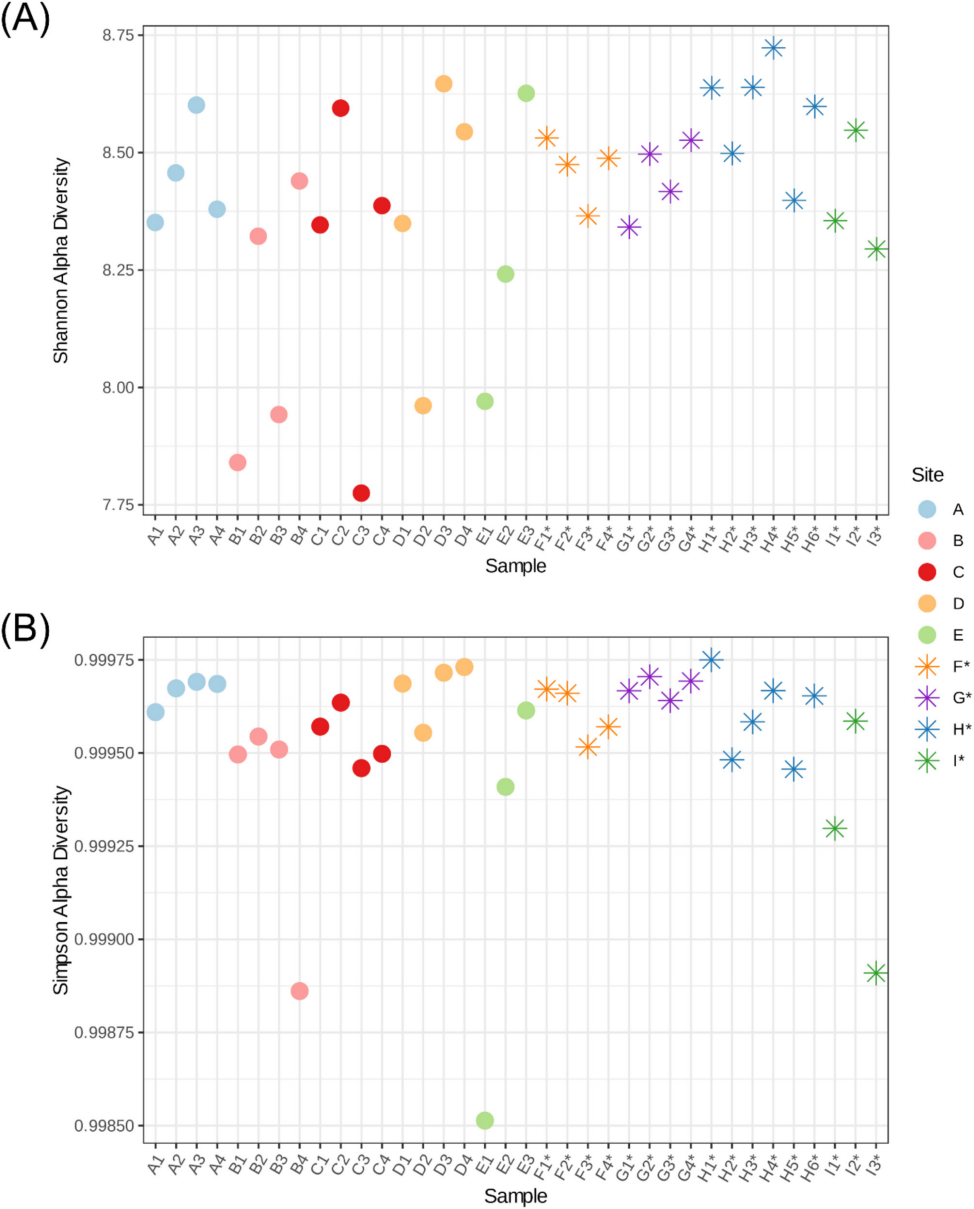
Diversity within each soil sample, as (A) Shannon and (B) Simpson alpha diversity indices. Each point along the *x*‐axis is a soil sample from an individual tree, and colors indicate sites. Diversity indices are shown on the *y*‐axis, where higher values represent greater microbial richness and evenness within the sample's microbial community.

Soil microbiomes of trees close to each other were similar according to Bray–Curtis NMDS, with the least variation at site A (Figure 5). Three major groups are evident: urban California samples (sites A‐E), wild California samples (sites F*–H*), and Hvar samples (site I*). Microbial composition significantly differed between wild and urban sites according to distance matrix ANOVA (*p* = 0.001). Shannon and Simpson indices quantify alpha diversity of detected microbes, with the Simpson measure weighing abundant microbes highly within the sample. Most (86%, 31) samples had Shannon indices in the 8.25–8.75 range, and the other five (D2, E1, C3, B3, and B1) were between 7.25 and 8.25 (Figure 6).

**FIGURE 7:**
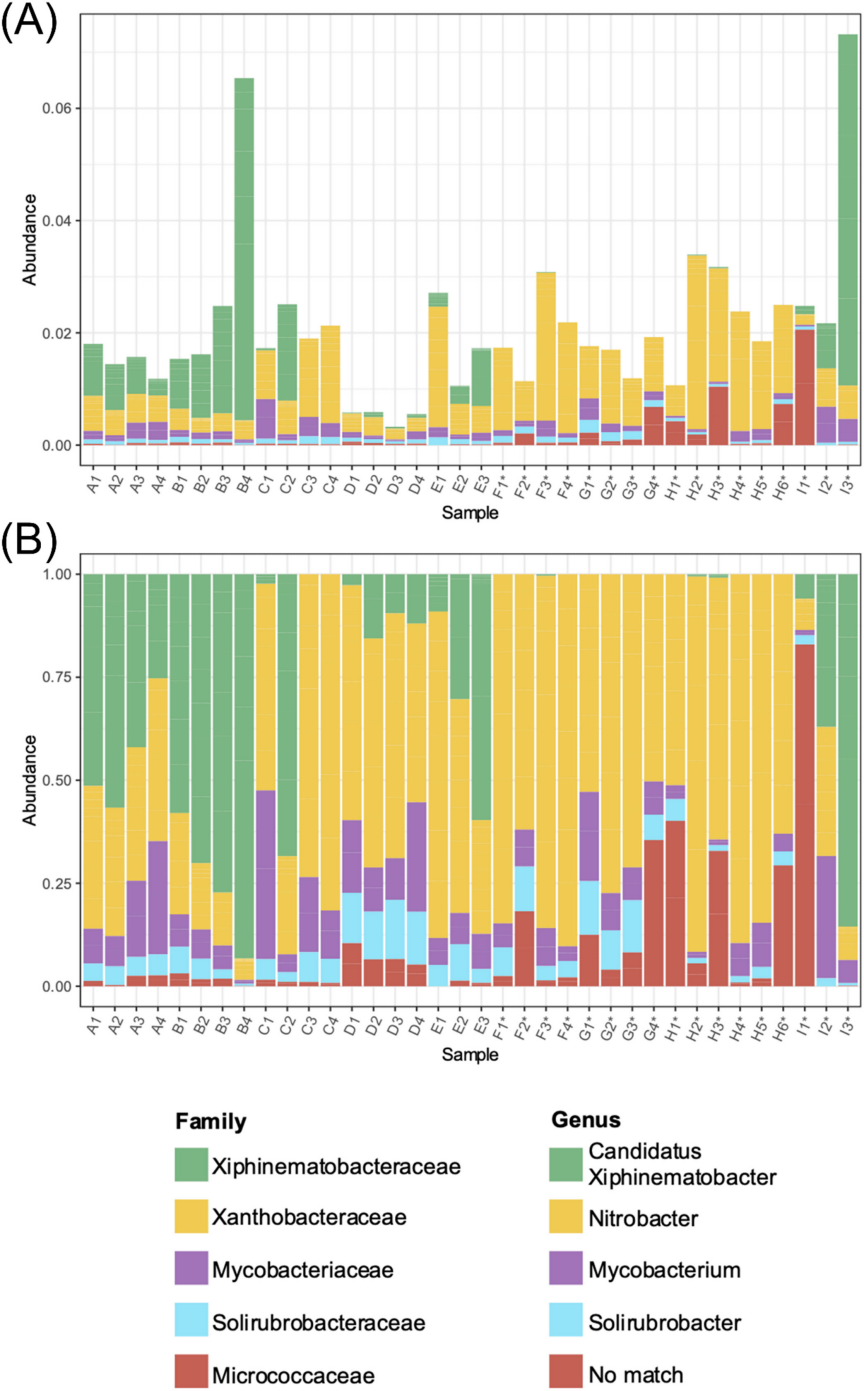
The 20 most abundant amplicon sequence variants (ASVs) in 16S rRNA by soil sample. All ASVs in a family belonged to the same genus, as indicated by shared colors. Bar heights represent abundances relative to sequences in (A) all samples and (B) within individual samples. ASVs of genera not identified in the SILVA database are shown as “No match.”

Micrococcaceae, Mycobacteriaceae, Solirubrobacteraceae, Xanthobacteraceae, and Xiphinematobacteraceae were present among the 20 most abundant amplicon sequence variants (ASVs) per sample (Figure 7A,B). Genera that could be determined included *Candidatus xiphinematobacter* of the Xiphinematobacteraceae family, *Mycobacterium* of the Mycobacteriaceae family, *Nitrobacter* of the Xanthobacteraceae family, and *Solirubrobacter* of the Solirubrobacteraceae family (Figure 7C,D). Wild California samples were characterized by abundant *Nitrobacter* (43%–89% of top 20 ASVs) and low *Candidatus xiphinematobacter* abundance (below 5%). Conversely, over 50% of the top 20 ASVs in A2, I3*, E3, C2, and all site B samples were *Candidatus xiphinematobacter*. Hvar samples varied in microbiome composition: I1* was the only sample dominated by Micrococcaceae of unidentified genera; I2* was evenly split between *Mycobacterium*, *Nitrobacter*, and *Candidatus xiphinematobacter*; and I3* was dominated by *Candidatus xiphinematobacter*.

### 3.5 Comparison of Concatenated and Barcode‐Specific Phylogenetic Trees Using Leaf DNA

**FIGURE 8:**
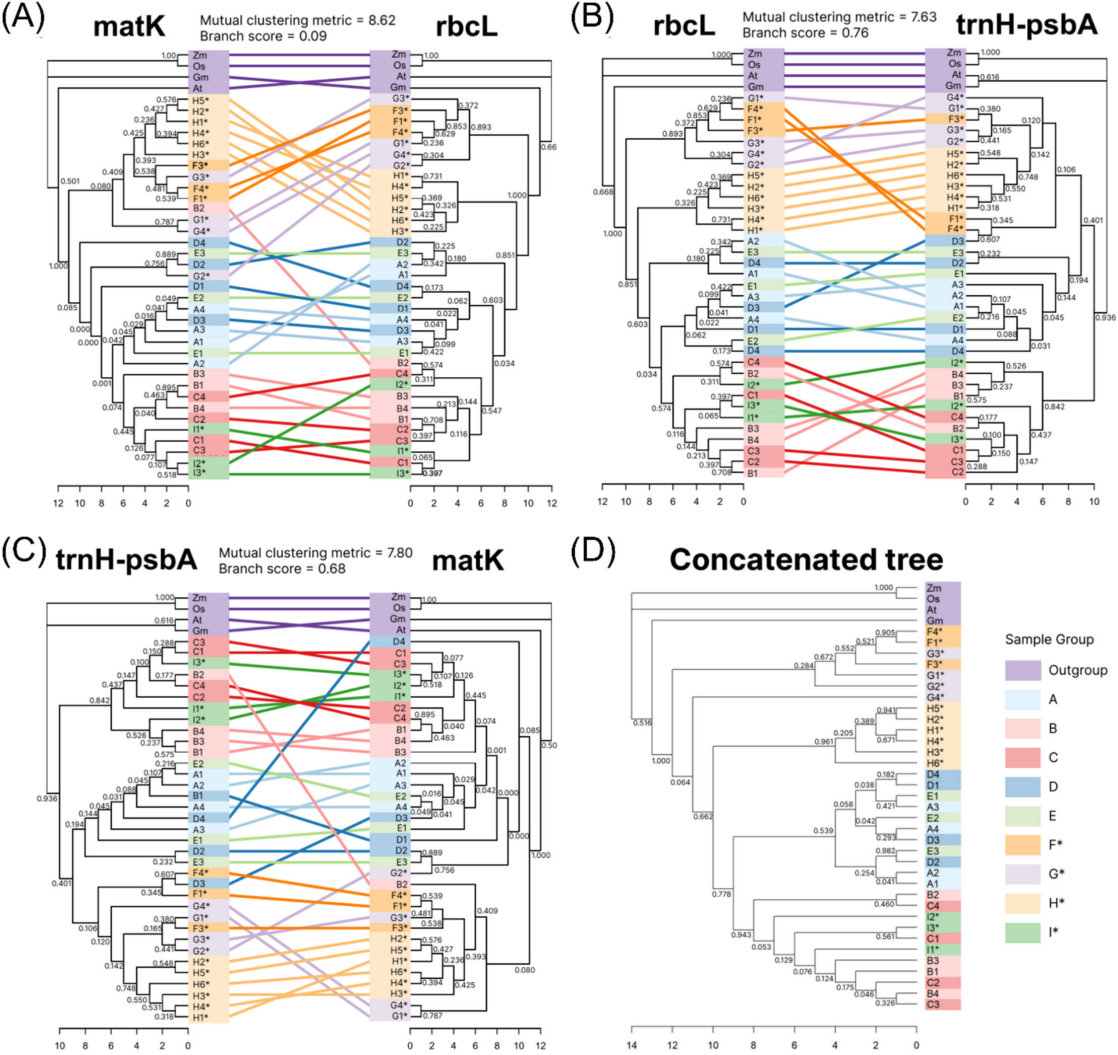
Individual and concatenated barcode phylogenies. Similarities between phylogenetic trees are compared between (A) *matK* and *rbcL*, (B) *rbcL* and *trnH‐psbA*, and (C) *trnH‐psbA* and *matK*, and (D) one phylogeny was determined using concatenated *matK*, *rbcL*, and *trnH‐psbA* sequences. Lines link corresponding samples, and colors represent sampling sites. *Zea mays* (Zm), *Oryza sativa* (Os), *Glycine max* (Gm), and *Arabidopsis thaliana* (At) were used as outgroups. Mutual clustering metrics and branch scores are indicated above each tanglegram to quantify similarity and dissimilarity between phylogenies, respectively. Branching patterns indicate relatedness, where samples with fewer nodes between them are more closely related, and branch lengths correlate with sequence differences.

In each phylogenetic tree, outgroups diverge before three major *Arbutus* clusters are formed: F*, G*, and H*; A, D, and E; B, C, and I* (Figure 8). F*/G*/H* clusters diverge from the other two with strong bootstrap support (BS > 0.85) in the *rbcL* and *matK* trees, while *trnH‐psbA* and the concatenated tree strongly support B/C/I* divergence (BS > 0.93). All individuals in the H* site consistently form their own clade across all phylogenetic trees, although only the *rbcL* tree exhibits strong bootstrap support (BS > 0.85). The *trnH‐psbA* tree has the most distinct clades of the three‐site clusters mentioned, while *rbcL* and *matK* trees show more divergence: F* and G* in *rbcL* and concatenated trees diverge first (BS = 1), and D4 in *matK* diverges before the clade containing clusters A/D/E and B/C/I* but with low bootstrap support (BS = 0.085). Outlying samples include G2* and B2 in *matK* clustering with A/D/E and F*/G*/H* respectively, along with D3 clustering with F*/G*/H* in *trnH‐psbA*. Mutual clustering metrics (Smith 2020) were used to compare tree topology between barcodes, while branch scores (Kuhner and Felsenstein 1994) compared both topology and branch length. The *matK* and *rbcL* trees are most similar (mutual clustering metric = 8.62; branch score = 0.092). The *trnH‐psbA* tree differed most from the *rbcL* tree (mutual clustering metric = 7.63; branch score = 0.76) and exhibited shorter branch lengths overall.

BLAST was used to tentatively identify species from each sequence. Except for G1*, G2*, G4*, and D2, all sequences yielded *A. unedo* in the top 10 species results with greater than 87% identity, sorted by E value. G1*, G2*, and G4* *matK* sequences match *Arctostaphylos uva‐ursi* best, but still have matches for *A. unedo* with scores above 1100, identity matches above 97%, and E values at approximately zero. *rbcL* and *trnH* sequences for these individuals yield *A. unedo* as at least one of the top three species matches except G1* *rbcL*, for which *A. unedo* is the fifth best match. The D2 *trnH* sequence yields *A. unedo* as the third best match, but with an E value of 2.00E−23 and 82.97% identity, which is similar to the top two best matches having an E value of 2.00E−42 and 85.71% identity, and E value of 2.00E−32 and 80.37% identity, respectively. Other common results included *A. menziesii* (native to North American western coast) and *A. andrachne* (native to Mediterranean region).

## 4 Discussion

We utilized *rbcL*, *matK*, and *trnH‐psbA*, three standard plant barcodes from plastid genomes, to derive taxonomic relationship among morphologically distinguishable planted and wild *Arbutus* individuals distributed across a relatively large geographic range. Despite the low resolution of barcodes and branch‐specific differences between phylogenetic trees, all barcodes distinguished between the same overall groups by both geography and planted vs. wild status. Sampling sites were geographically divided into Southern California, Northern California, and Hvar (Figure 1). We expected phenotypic variation to correlate with plant genotypes and geographic distribution within and between sites, such that planted, clustered urban trees and their manicured soil show the least variation in morphology (tree, leaf, and fruit), chemistry, microbiome composition, and barcode sequences. As the geographical distances increase, variation in phenotypes and barcode sequences were expected to increase; notably, low variance compared with other wild tree groups was expected among Croatian island natural population trees due to a lower sample size with proximity among individual trees, and geographical distance from other populations. Because these trees were not deliberately planted or maintained, they were still expected to display a larger variation than closely clustered, urban coastal Southern California planted trees and associated soil.

Of the Southern California sites (A–G), B, C, and D are geographically closest, A and E are located a similar distance to the east of the three clumped sites, and F* and G* are the easternmost sites and distant from the others. Northern California site H* is a considerable distance (about 925–1000 km) away from the Southern California sites, and Hvar site I* samples were collected on an island from a separate continent (Figure 1, Table 1). The three closest‐lying Southern California sites (B, C, and D) and A and E are in urban areas and individual plants sampled at these sites are likely maintained for their ornamental value, as evidenced by the manicured nature of their surroundings. All other sites are wild, as plants grow spontaneously without deliberate placement or maintenance. These sampling sites allowed for the effects of environmental variation, including both geographical locations and site management status (i.e., manicured or wild) to be determined for within‐ and between‐population comparisons.

The grouping of similar sequences was expected to be consistent across the barcodes based on sample location, population density, and status as planted vs. wild populations, with the use of evolutionarily distant species as controls (Syamsuardi and Okada, H. 2002; Leimu et al. 2006). Due to selection for desired uniform esthetics, manicured gardens or urban sites' plants generally have less genetic diversity than wild plant counterparts from untamed landscapes (Gao et al. 2017; Huang et al. 2019; Tourvas et al. 2023), although there are exceptions as some cultured and wild plants can have similar diversity (Sousa‐Junior et al. 2023). Greater geographic distance was expected to correlate with barcode sequence divergence both within and between sites due to environmental differences, especially among wild *Arbutus* from larger populations with decreased inbreeding (Keller and Waller 2002; Angeloni et al. 2011). Four main groups were resolved by all barcodes and concatenation, mostly expectedly diverging in the following order (Figure 8): (1) outgroups (soybean, corn, rice, and *Arabidopsis*); (2) wild California *Arbutus* (F*/G*/H*); (3) Southernmost and Northernmost planted California *Arbutus* (A/D/E); (4) other planted California and wild Hvar *Arbutus* (B/C/I*). The early outgroup divergence was consistent among all phylogenies with strong bootstrap support, as expected from their relatively distant relation to *Arbutus*.

The wild California group (F*/G*/H*) was most morphologically and geographically diverse among US samples. All fruits in this group were small (roughly 3 cm in diameter), round, and with smooth skin. The wild Northern California individuals (H*) consistently formed an exclusive clade regardless of barcode, as expected from their distinct phenotype compared with the wild Southern California individuals (F*/G*). These northern trees are the tallest and most robust of all sampled sites, with well‐established tree canopies and dark green fruit clusters. Their relatively northern location, spanning 65 km, exposes them to the coldest climate and greatest annual rainfall of sampled trees. Although belonging to this same group, the wild *Arbutus* in Southern California are smaller and bush‐like, with rounded leaves. The southernmost of these (F*) bore light green fruit, while the others (G*) bore yellowish fruit.

The third and fourth groups include all planted individuals and the wild Hvar individuals. *matK*, *rbcL*, and concatenation (Figure 8), place these groups in the same clade, as consistent with their morphological similarities, including both tree‐like single trunks and bush‐like multi‐branch trunks. Except for Hvar trees (I*), all individuals in this group have dark green leaves with pointed tips and red‐orange mature fruits approximately 5–6 cm in diameter, notably larger than those of wild populations. Trees in the third group (A/D/E) were the southernmost and northernmost planted populations but were still restricted to Southern California within a range of 33 km. The fourth group consisted of a tight cluster of planted trees (B/C) within 4 km, and the distant wild Hvar population (I*). Phylogenetic branch lengths are shortest in this group, indicating high sequence similarity. This is expected among the B/C trees considering their short geographic distance and almost indistinguishable morphology. Inclusion of Hvar samples is unexpected, considering its distant location and status as a wild island population, in contrast to the planted trees in this group; however, the microclimates and annual rainfall of Hvar and coastal Southern California are notably similar (Croatian Meteorological and Hydrological Service 2024; National Weather Service 2024), so related selective pressures may explain this topology.

Although these four main groups are consistent across barcodes, the topology of individuals within these groups varies. Nodes have been rotated for better alignment across phylogenetic trees without affecting topology or branching pattern. For example, *matK* trees in Figure 8A,C are equivalent despite differences in sample position. Branching patterns reveal that some individuals depart from the four main groups: *matK* groups G2* with the northernmost and southernmost planted trees (A/D/E) and B2 with the wild California populations (F*/G*/H*); *trnH‐psbA* groups D3 with the same wild California group (F*/G*/H*). The *rbcL* phylogram is overall relatively unbalanced compared with *matK* and *trnH‐psbA*, suggesting slower diversification in the *rbcL* region among wild California populations compared with other populations. Therefore, *rbcL* might exhibit greater resolution than other barcodes for resolving relationships among planted, closely related individuals. *rbcL* encodes the large subunit of rubisco, which is the most abundant protein in leaves and is critical for photosynthesis (Hauser et al. 2015). While the sample size limits the ability to perform purifying selection analysis, purifying pressures may have slowed diversification in the wild Southern California population, while positive artificial selection could speed *rbcL* diversification among planted individuals. Despite these differences, the main groupings are largely consistent across all barcodes. The three standard plant barcodes resolved trees as expected from geographic location and plant vs. wild population phenotypes, with some minor differences. Variation in topologies between the barcodes implies insufficient resolution for any one barcode to distinguish within‐group relationships; therefore, the similarities between all three barcodes provide the most reliable inferences. All trees were sufficient to distinguish only between the major three groups within the closely related *Arbutus* individuals. Employing multiple barcodes such as these is a common strategy to obtain greater reliability than a single barcode could provide (Yuan et al. 2015; Srivastava et al. 2022; Fu et al. 2024). In cases with resource constraints, this three‐barcode strategy provides a practical avenue for preliminary evidence that can validate further hypothesis building, particularly if followed by more comprehensive methods when appropriate.

Fruit chemistry results agree with only some of the observed morphological variance (Figures 2 and 3). Mineral, crude protein, and crude fiber content clusters all Hvar (I*) fruits together, suggesting similarity due to the shared microenvironment and microclimate. B/C fruits have similar chemical compositions, consistent with their narrow range of 3 km and morphological resemblance. Surprisingly, one of the planted populations' fruits (D) from the maintained urban site with smallest trees does not cluster with any other samples, despite similarities in location and morphology with other planted trees. While fruit chemistry is different, no other analysis depicts site D as an outlier. The small fruits from wild populations in Hvar and Southern California (I*/F*/G*) cluster together. Although the wild Northern California populations (H*) also bear small fruit, they cluster with the Southernmost, planted samples (A). Northern California trees have large leaves resembling those of the planted populations more than the wild ones, and otherwise exhibit the most distinct morphological phenotypes: the tallest single‐trunk trees with broad canopies and small, smooth, dark green fruit. The moisture in fruits seems to not correlate with any other observed morphological or geographical patterns. Fruit content varies by maturity and is subject to environmental, seasonal, and genetic factors (Tulipani et al. 2011; Tartaglia et al. 2023), possibly explaining its only partial alignment with location, morphology, and genetic similarities.

Microbes living on and in plants and plant‐associated soil can play important roles in host nutrition, development, and immunity (Carvalhais et al. 2013; Sahu et al. 2019; Korenblum et al. 2020; Panda and Das 2024). How plant evolution and physiology interact with soil microbial and chemical composition is mostly unknown. Geographic location, topography, and rainfall variations can affect soil microbiomes by selecting for or against certain taxa over both long and short time periods (Silva et al. 2022; Vasar et al. 2022; Wang et al. 2022). pH and annual precipitation are among the most influential abiotic factors influencing bacterial communities, with soil moisture playing a lesser role (Bahram et al. 2018). Microbiome composition has been associated with plant mechanisms of sensitivity and resistance to biotic and abiotic stressors (Alavi et al. 2013; Panda and Das 2024). We complement our phylogenetic analysis of *Arbutus* with bulk soil microbial profiling, using 16S rRNA sequencing to better understand how the microbial association with *Arbutus* trees is affected by factors such as topography, climate, tree morphology, and level of human interaction (planted vs. wild populations).

Besides biotic interaction between plants and the environment, abiotic factors, often combined with the human influence, also play an important role in genetic diversity. Bulk soil sample composition can help characterize ecologically relevant soil conditions including other nearby plants and human influence. Wild tree populations are part of diverse, uncontrolled systems, while planted populations are often artificially maintained with minimal surrounding plant diversity and nutrient‐rich commercial soil. Reduced natural selection pressures due to human maintenance may suppress the need for adaptive traits in response to changing conditions. For instance, planted populations have reliable water and nutrient sources with minimal competition. Precipitation at the wild Northern California sites is most variable, with dry summers and rainy winters. Compared with the Southern California sites, the Northern California sites receive roughly five times the precipitation, and the Hvar sites receive three times the precipitation yearly (Croatian Meteorological and Hydrological Service 2024; National Weather Service 2024), thus impacting soil conditions and favoring bacteria suited to local conditions (Bahram et al. 2018). At the time of sampling, the Southern California trees received very little rainfall, highlighting the effect of maintenance on water supply (Supporting Information S1).

Nearby *Arbutus* trees' soil tends to share similar chemical compositions according to hierarchical clustering of characteristics including lime buffer capacity, pH, percent base saturation, total organic carbon, and mineral content (Figure 4). As expected, soil from individuals in sites A (planted, Southern California), G* (wild, Southern California), and H* (wild, Northern California) cluster by site, which confirms site grouping based on soil attributes. Because secondary metabolites not included in our analysis undergo selective pressures and vary under different climates and urban conditions (Charlop‐Powers et al. 2016; Bont et al. 2020), their quantification would further inform investigation of environmental adaptation and taxonomic differentiation. Known seasonal and weather‐based variations by time of collection, particularly among wild *Arbutus* populations, may explain patterns that diverge from expectation: for example, soil from one Hvar tree (I1*) clusters with wild sites (F*, H*), all of which have sandy texture. Surprisingly, two Southern California planted *Arbutus* soil samples (C1 and D1) clustered with one nearby Southern California wild site (G*). The continent of origin and management status are reflected in the distinct NMDS clustering of soil microbes from (1) all planted trees, (2) all wild California trees, and (3) all wild Hvar trees. I2* and I3* soil also had similar chemical composition (Figure 5), highlighting chemical characteristics' association with certain microbes (Gul et al. 2015; Tian et al. 2017; Philippot et al. 2023). *Nitrobacter* was abundant (43%–89% of top 20 ASVs) in all wild California microbiomes (Figure 7) and plays a role in nitrification by oxidizing nitrite to nitrate. High potential nitrite oxidation has been correlated to abundant *Nitrobacter*‐like bacteria, high organic carbon content, and untilled land (Attard et al. 2010), all of which are features of the soil sample of wild *Arbutus* populations. By contrast, *Candidatus xiphinematobacter* exhibited relatively low abundance in wild California microbiomes but comprised over 50% of the top ASVs in A2, I3*, E3, C2, and all site B samples. Bacteria in this family are endosymbionts of the dagger nematode (*Xiphinema*), a root parasite (Vandekerckhove et al. 2015). Soil from wild California populations exhibited a comparatively low presence of *Candidatus xiphinematobacter*, suggesting that there may be a larger presence of dagger nematode near planted *Arbutus*ASVs of the Micrococcaceae family were only abundant (over 10% of top 20 ASVs) in soil associated with wild trees (F2*, G4*, H1*, H3*, H6*, I1*) (Figure 7). Micrococcaceae is correlated with microbial nitrogen limitation in oligotrophic ecosystems with low nutrient levels. Nutrient limitations in soil impact the metabolism of microbes and plants, resulting in competition within and between them (Xu et al. 2015). Microorganisms are also key to nutrient utilization by plants, as they decompose organic matters bound to soil particles and minerals (Cui et al. 2022). Mycobacterium, another abundant ASV, has also been reported as the second most abundant endophytic bacteria in *A. unedo* (Martins et al. 2022). Notably, while some of the microbial content is group and site‐specific, multiple variables influence bulk soil composition, including seasonal, weather, elevation, and spatio‐temporal variation.

We anticipated that geographically close planted trees would have similar genotypes and phenotypes, and vice versa. DNA barcoding enables relatively quick and affordable diagnostic identification of species present in specific locations with immediate conservation and environment‐related implications. Therefore, this method was developed as an aid to the taxonomic identification of species (CBOL Plant Working Group 1 et al. 2009; Mahadani et al. 2022). Although these barcodes are often used in other plant taxa (CBOL Plant Working Group 1 et al. 2009; Tripathi et al. 2013; Bieniek et al. 2015; Cabelin and Alejandro 2016), their individual low resolutions preclude strong inferences (Dong et al. 2012); however, several barcodes taken together can provide between‐group distinction based on overall commonalities between barcode results. These distinctions, supported by all barcodes, imply targeted genetic distances between groups of planted and wild *Arbutus* individuals in a wide geographic range. Several factors can hinder reliable species identification using standard plant DNA barcodes (Kress 2017). Such issues can arise among woody species like *Arbutus* trees with long generation times and/or slow mutation rates and in groups with evidence of recent radiation: sufficient time since speciation is required for point mutations or genetic drift, resulting in a set of genetic characters that “group” or outgroup unique individuals from other species. In phylogenetic evaluation, barcode sequences are similar among related taxa or species in clades where speciation has been very recent. Moreover, determining fine‐scale speciation in plants can be challenging because species boundaries are not well defined (Fazekas et al. 2008), which is the case in our Southern‐to‐Northern *Arbutus* boundaries, confounded by a relatively large phenotypic variation, including leaf and fruit morphological complexity observed among our planted and wild *Arbutus* populations. The use of only plastid barcodes also limits phylogenetic analysis because these loci are generally uniparentally inherited (Corriveau and Coleman 1988). Furthermore, while trnH‐psbA is highly variable and potentially has the highest resolution of the three barcodes (Kress et al. 2005), it often contains tandem repeats (Hao et al. 2010) that can challenge proper alignment and result in biases. Hence, a complexity of taxonomic groups cannot be solved using one or few barcodes, since such tree groups may result from recurrent ecotypic taxonomic origins. For example, in *Solanum* sect. *Petota* (wild potatoes), *ITS*, *trnH‐psbA*, and *matK* regions showed too much intra‐specific variation and lacked sufficient polymorphism (Spooner 2009). Also, the universal barcodes are not effective in Indian *Berberis* and two other genera, *Ficus* and *Gossypium*. Even the most promising plant DNA barcode loci—*ITS* from the nuclear genome, and *trnH‐psbA*, *rbcL*, and *matK* from the plastid genome—failed to resolve species in these plant groups (Roy et al. 2010). One of the proposed alternative approaches is indel polymorphism method as a species‐level marker tested in Citrus (Mahadani and Ghosh 2014). When combined with plastid barcodes, nuclear markers such as microsatellites and *ITS*, could represent inheritance from both parents at higher resolution and yield a more reliable and comprehensive analysis within species (Joshi et al. 1999; Zimmer and Wen 2015; Fu et al. 2024). Methodologies such as RAD‐seq or genotyping‐by‐sequencing cover multiple loci across the genome simultaneously, providing a possible intermediate between our three‐barcode analysis and cost‐prohibitive whole‐genome sequencing (Zimmer and Wen 2015).

Similarities in barcode sequences make it challenging to identify all samples as *A. unedo* confidently. While BLAST overwhelmingly yielded *A. unedo* as a top result for most sequences, *A. uva‐ursi* was a better match for *matK* sequences from G1*, G2*, and G4*. The distinct morphology of G* trees, with smooth fruit and small round leaves, resembles *A. uva‐ursi* . However, *A. unedo* was a top result for *rbcL* and *trnH‐psbA*, matching better than *A. uva‐ursi* for five of the six sequences. Overall common results across all barcodes and samples included *A. menziesii* and *A. andrachne* , which bear morphological resemblance to *A. unedo* with reddish bark, yellow‐to‐red round bumpy fruit, and dark green oblong leaves. *A. menziesii* is native to California, and *A. andrachne* is closely related to *A. unedo* . Discriminating between these similar species is further complicated by potential hybridization and poorly defined species boundaries. Markers such as microsatellites should offer better resolution for assessing both within‐species diversity (Gomes et al. 2013; Ribeiro et al. 2017) and accurate identification across species (Mathew et al. 2020).

Notable morphological variations of trees, leaves, and fruits (Figure 2) led to difficulty in determining the species of sampled plants at collection. Differences in environment have the potential to lead to morphological differences in plants of the same or closely related species (Møller and Shykoff 1999; González and Gianoli 2004) (Supporting Information S1). Specifically, soil composition (Figure 4) and temporal variation in sample collection could have affected results, while chemical composition of *Arbutus* fruits have been found to differ at different fruit ripening stages (Oliveira et al. 2011). Differences in precipitation affect soil microbiome, with more arid environments displaying decreased functional diversity (Tripathi et al. 2017). While our data demonstrated overall agreement among standard plant barcodes relative to geo‐distribution and plant morphology, a large sample size is recommended to further detect potential speciation signatures with higher resolution. Furthermore, when practical, nuclear markers or coding and noncoding nuclear regions would provide more reliable results. Our data provide a high‐level understanding of planted and wild *Arbutus* phylogeny from two continents, clearly inviting further investigation using more comprehensive methodologies informed by our preliminary analyses.

## Author Contributions

Flannery McLamb: Analyzed data, wrote and revised the paper. Armando Vazquez: Performed the research, analyzed data, wrote and revised the paper. Natalie Olander: Analyzed data, wrote and revised the paper. Miguel F. Vasquez: Performed the research, revised the paper. Zuying Feng: Analyzed data, wrote and revised the paper. Niharika Malhotra: Analyzed data, wrote and revised the paper. Liisa Bozinovic: Performed the research, analyzed data, wrote and revised the paper. Karen Najera Ruiz: Analyzed data, revised the paper. Katherine O'Connell: Performed the research, revised the paper. Joe Stagg: Performed the research, wrote and revised the paper. Goran Bozinovic: Conceived and designed the project, performed the research, analyzed the data, wrote and revised the paper.

## Conflicts of Interest

The authors declare no conflicts of interest.

## Significance Statement

Taxonomic analysis of *Arbutus* plants reveals similar fruit chemistry and morphology within a narrow geographical range, while bulk soil bacterial content defines three distinct clusters reflecting planted versus wild trees and continent of origin. Three barcodes resolved four main groups, with the relatively unbalanced *rbcL* phylogram suggesting slower diversification among wild California wild populations and greater resolution than *matK and trnH‐psbA* within planted trees.

## Supporting information


**Data S1** Supplementary Material.

**Table S1** Sources of barcoding target sequences for selected outgroups: *Glycine max* , *Zea mays* , *Oryza sativa* , and *Arabidopsis thaliana* .
**Table S2.** 16 s rRNA read counts per soil sample after sequencing, filtering, merging paired‐end reads, and removing chimeras.
**Table S3.** Fruit chemistry raw data values as percentages of fruit as sampled or as dry‐matter.
**Table S4.** Soil chemical analysis raw data values from soil samples pooled by individual tree.

## Data Availability

Raw sequencing reads are available on the SRA. Leaf tissue DNA accession numbers are SAMN41150687‐SAMN41150721 under BioProject PRJNA1106814, and 16S rRNA accession numbers are SAMN41135305‐SAMN41135340.
